# Elevated seminal plasma estradiol and epigenetic inactivation of *ESR1* and *ESR2* is associated with CP/CPPS

**DOI:** 10.18632/oncotarget.24714

**Published:** 2018-04-13

**Authors:** Nils Nesheim, Stuart Ellem, Temuujin Dansranjavin, Christina Hagenkötter, Elena Berg, Rupert Schambeck, Hans-Christian Schuppe, Adrian Pilatz, Gail Risbridger, Wolfgang Weidner, Florian Wagenlehner, Undraga Schagdarsurengin

**Affiliations:** ^1^ Clinic of Urology, Pediatric Urology and Andrology, Justus Liebig University, Giessen, Germany; ^2^ Working Group Epigenetics of the Urogenital System, Clinic of Urology, Pediatric Urology and Andrology, Justus Liebig University, Giessen, Germany; ^3^ Department of Anatomy and Developmental Biology, Monash University, Clayton, Victoria, Australia; ^4^ Department of Physiology, Biomedicine Discovery Institute, Monash University, Clayton, Victoria, Australia

**Keywords:** chronic prostatitis/chronic pelvic pain syndrome (CP/CPPS), liquid biopsies, estradiol, estrogene receptor, epigenetic inactivation

## Abstract

Chronic prostatitis/chronic pelvic pain syndrome (CP/CPPS) is associated with urinary tract symptoms and hormonal imbalances amongst others. The heterogeneous clinical presentation, unexplored molecular background and lack of prostate biopsies complicate therapy. Here, using liquid biopsies, we performed a comprehensive translational study on men diagnosed with CP/CPPS type III (*n* ***=*** 50; median age 39.8, range 23–65) and age-matched controls (*n* ***=*** 61; median age 36.8, range 20–69), considering biochemical parameters of blood and ejaculates, and epigenetic regulation of the estrogen receptor genes (*ESR1* and *ESR2*) in leukocytes isolated from blood (systemic regulation) and in somatic cells isolated from ejaculates (local regulation). We found elevated 17β-estradiol (E_2_) levels in seminal plasma, but not in blood plasma, that was significantly associated with CP/CPPS and impaired urinary tract symptoms. In ejaculated somatic cells of CP/CPPS patients we found that *ESR1* and *ESR2* were both significantly higher methylated in CpG-promoters and expressionally down-regulated in comparison to controls. Mast cells are reported to contribute to CP/CPPS and are estrogen responsive. Consistent with this, we found that E_2_ –treatment of human mast cell lines (HMC-1 and LAD2) resulted in altered cytokine and chemokine expression. Interestingly, in HMC-1 cells, possessing epigenetically inactivated *ESR1* and *ESR2,* E_2_ –treatment led to a reduced transcription of a number of inflammatory genes. Overall, these data suggest that elevated local E_2_ levels associate with an epigenetic down-regulation of the estrogen receptors and have a prominent role in CP/CPPS. Investigating E_2_ levels in semen could therefore serve as a promising biomarker to select patients for estrogen targeted therapy.

## INTRODUCTION

The prostate is particularly susceptible to disease, with ∼90% of men developing histological benign prostatic hyperplasia (BPH) by the age of 80 [1], ∼11.6% of all men developing prostate cancer (PCa) during their lifetime [2], and up to 16% developing chronic prostatitis/chronic pelvic pain syndrome (CP/CPPS) [3]. CP/CPPS generally receives less attention and remains poorly understood, even though it has a high disease burden. CP/CPPS is characterized by pelvic pain, ongoing urinary tract symptoms without detectable pathogens or identifiable aetiology, and various other signs and symptoms, assigning the syndrome to the class of chronic inflammatory and perhaps autoimmune disorders [4].

There is currently no reliable biomarker for CP/CPPS. Instead, classification as inflammatory (IIIa: leukocytes) or non-inflammatory (IIIb: no leukocytes) is based on upon the presence or absence of leukocytes in expressed prostatic secretions or post prostatic massage urine [5]. However, this classification does not have implications for differential treatment [6], nor does the presence of leukocytes in prostatic secretions correlate with severity of symptoms [7]. Instead, it was the introduction of a multimodal treatment system (UPOINT) with symptom-specific patient stratification that has led to significant advances in disease management [8, 9], indicating that multiple disease entities could be concealed behind the diagnosis CP/CPPS. Nevertheless, the lack of reliable biomarkers and well-established patho-mechanisms are the reasons why the syndrome is treated in a phenotype-directed manner. The high prevalence and morbidity of CP/CPPS, as well as the possible link to PCa and/or BPH upon aging, stresses the need for a deeper understanding of the underlying mechanisms.

Steroid sex hormones, particularly testosterone and estradiol (E_2_), play key roles in the development and maintenance of the reproductive systems. The synthesis of estrogens from androgens is catalyzed by aromatase. Estrogen action in the male is viewed in at least two different ways: systemic endocrine effects acting through the pituitary gland to indirectly lower androgens and local effects that directly target prostate tissue by estrogen receptors [10]. Androgen and estrogen signaling plays a significant role in normal and abnormal growth of the prostate gland [10]. Studies examining the steroid hormones in CP/CPPS, however, are equivocal, reporting either elevated systemic testosterone and unchanged estradiol levels [11], or elevated systemic estradiol levels, but unchanged testosterone levels [12]. In the latter, it is interesting to note that this patient cohort also had an elevated body mass index, so adipose aromatase activity could account for the elevated systemic estradiol levels.

Prostate biopsies in patients with CP/CPPS are not routinely performed, which has led to the development of multiple animal models such as experimental autoimmune prostatitis (EAP). These include mouse models of chronic prostatic inflammation induced by immunization with prostate antigen (PAg) [13], rat models with chronic inflammation induced by combined testosterone and E_2_ treatment [14], or a transgenic mouse model with aromatase (ARO+) over-expression leading to chronic prostatitis and prostate pre-malignancy with increased mast cell infiltration [15]. Further studies have subsequently linked mast cell infiltration and prostatic estrogen dominance to prostate cancer [16]. Elevated tryptase levels were also present in the expressed prostatic secretions (EPS) of CP/CPPS patients [17], underscoring the clinical significance of mast cells and drawing attention to estrogen as a potential mediator of both PCa and CP/CPPS.

At least two compounds targeting estrogen signaling have been studied for treatment of CP/CPPS. Patients were shown to profit by treatment of Mepartricin, an estrogen reabsorption inhibitor [18], but also from quercetin, a plant isoflavonoid with anti-estrogenic properties and a ∼9 fold higher affinity to estrogen receptor beta (ERβ, *ESR2* gene) than to ER-alpha (ERα, *ESR1* gene) [19–21].It is interesting to note that ERβ is a potent suppressor of inflammation in multiple tissues/organs, including the brain and bowel [22, 23]. Hence, an aberrant and increased prostatic ERα:ERβ ratio may contribute to CP/CPPS.

Administration of the histone deactelyase (HDAC) inhibitor MS-275 led to EAP attenuation in a rat model [24], highlighting the epigenetic dimensions of the inflammatory response as a possible target for epigenetic drugs. Our group recently reported epigenetic inactivation of CXCR4 (C-X-C motif receptor of the chemokine CXCL12/SDF1) in CP/CPPS patients’ liquid biopsies [25], showing that CP/CPPS is accompanied by systemic and organ-specific epigenetic changes. Here, we extend upon this and examine in a prospective analytical comparative study whether epigenetic aberrations of the sex hormone receptor genes *ESR1*, *ESR2* and *AR* (androgen receptor) occur in CP/CPPS and associate with the clinical phenotype. This study was approved by the Ethics Commission of the Medical Faculty of the Justus-Liebig-University Giessen (ethical votes, AZ.: 55/13; AZ.: 123/12) and all subjects provided written informed consent. To provide mechanistic insights for our findings in patients’ liquid biopsies, and to explore the role of mast cells and estrogen in CP/CPPS, we studied human mast cells and the influence of estrogen on their inflammatory profile. Overall, we provide new molecular insights into the chronification of prostatitis and demonstrate that seminal plasma estradiol levels and epigenetic state of estrogen receptor genes, respectively, may be a novel diagnostic tool for CP/CPPS patients that could be used to select patients for targeted therapy.

## RESULTS

### Increased concentration of 17β-estradiol in seminal plasma is associated with CP/CPPS and impaired urogenital tract symptoms

**Figure 1:**
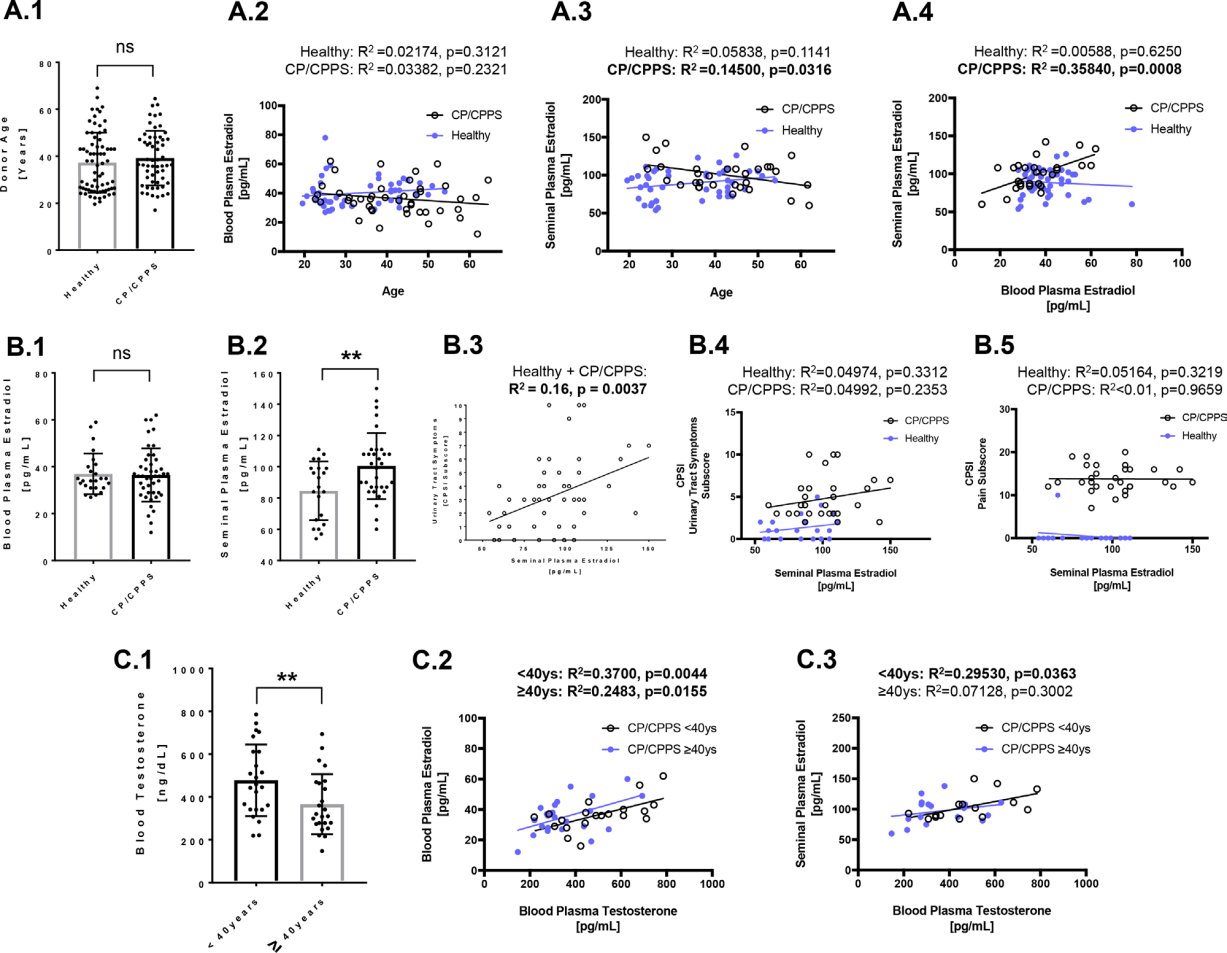
Characterization of analyzed cohorts with regard to estradiol (E_2_) and testosterone levels in blood and seminal plasma (**A**) Age-matched cohorts of healthy men and CP/CPPS patients (**A.1**) were analyzed regarding the interdependence of systemic (in peripheral blood) and local (in seminal plasma) E_2_ levels to age and to one another. In both, controls (blue filled circles) and patients (empty circles) blood E_2_ was not correlated to age (**A.2**). In patients, but not in controls, a moderate significant correlation was found between seminal plasma E_2_ and age (**A.3**). In patients, but not in controls, a highly significant correlation between seminal plasma E_2_ and blood E_2_ was found (**A.4**); (**B**) By comparing controls and patients with regard to systemic and local E_2_ levels, a significant increase of E_2_ were detected in seminal plasma of CP/CPPS men, whereas E_2_ levels in blood were similar (**B.1** and **B.2**). Striking, considering controls and patients together, a highly significant correlation between seminal plasma E_2_ and deterioration of urinary tract symptoms was found (**B.3**). However, in a separated analysis of controls and patients this correlation was less obvious (**B.4**). No correlation was found between the pain subscore and seminal plasma E_2_ (**B.5**); (**C**) Testosterone was routinely measured in CP/CPPS patients’ peripheral blood. Older patients (≥40 years) possessed significantly reduced testosterone levels than younger patients (<40 years) (**C.1**). Young as well as older patients exhibited a significant positive correlation between E_2_ and testosterone in blood (**C.2**). In young patients’ seminal plasma E_2_ and testosterone were also significantly correlated, whereas in older patients this correlation was absent (**C.3**). Abbreviations: ^**^p ≤ 0.01; ns: not significant (*p >* 0.05); CPSI: chronic prostatitis symptom index.

Whole blood and semen samples from CP/CPPS patients and healthy volunteers were analyzed in order to identify CP/CPPS associated systemic and local changes in sex hormone signaling (Figure 1). The median age of CP/CPPS patients was 39.76 years (range 23–65). As hormonal balance and imbalance, respectively, are age-dependent, an age-matched control cohort (median age 36.77, range 20–69) of healthy men without any preexisting urological conditions was also gathered (Figure 1A.1). By considering CP/CPPS patients and controls together as well as by analyzing them separately, we did not find a correlation between age and 17β-estradiol (E_2_) concentrations in blood plasma (Figure 1A.2). A low positive correlation between age and E_2_ in seminal plasma (R^2^ = 0.145, *p* = 0.0316) was found exclusively in the CP/CPPS patient group (Figure 1A.3). Interestingly, only CP/CPPS patients, but not healthy controls, exhibited a strong correlation between E_2_ levels in blood and in seminal plasma (R^2^ = 0.35840, *p* = 0.0008) (Figure 1A.4). CP/CPPS patients and controls did not differ in blood E_2_ levels (36.45 ± 1.71 versus controls: 36.96 ± 1.73 pg/ml; *p* > 0.05) (Figure 1B.1). However, E_2_ levels in seminal plasma were significantly increased in CP/CPPS patients compared to controls (CP/CPPS: 100.5 ± 3.72 versus controls: 84.57 ± 4.09 pg/ml; *p ≤* 0.01) (Figure 1B.2). Further, the seminal plasma E_2_ concentrations were analyzed in patients and controls with regard to the chronic prostatitis symptom index (CPSI), an evaluation system for the severity of CP/CPPS which comprises the subscores for urinary tract (voiding) symptoms, pain and quality of life. Increased E_2_ concentrations in seminal plasma correlated with impaired urinary tract symptoms, when CP/CPPS patients and controls were analyzed together (R^2^ = 0.16; *p* = 0.0037) (Figure 1B.3). However, this trend was less pronounced in the individual groups (Figure 1B.4). The quality of life and pain scores, on the other hand, were not correlated with E_2_ concentrations in seminal plasma (Figure 1B.5; a scatter plot for the CPSI pain subscore and E_2_ is shown; see also Supplementary Figure 1).

Testosterone was routinely measured in the blood of CP/CPPS patients: Older patients (≥40 years) possessed significantly lower systemic testosterone levels compared to younger patients (≥40y: 315 ± 140 versus <40y: 458 ± 288 pg/ml; *p* = 0.010) (Figure 1C.1). A significant positive correlation between blood testosterone and blood E_2_ was found in both age groups, whereby the correlation was more pronounced in younger patients (<40y: R^2^ = 0.37, *p* = 0.0044; ≥40y: R^2^ = 0.2483, *p* = 0.0155; Figure 1C.2). In younger patients, also a significant positive correlation between blood testosterone and E_2_ in seminal plasma was found (R^2^ = 0.2953, *p* = 0.0363; Figure 1C.3).

### Semen samples are a source of inflammatory markers of CP/CPPS

Ejaculates of CP/CPPS patients and healthy controls were compared with regard to routine semen parameters (Supplementary Figure 1). In particular, significant impairments in ejaculate volume, sperm concentration, total number, motility, morphology and vitality of sperm cells and pH-value could be evaluated in CP/CPPS patients in comparison to healthy controls (Supplementary Figure 1). These subsequent results confirmed our previously described and discussed observations in CP/CPPS patients [25] and emphasized again the relation between CP/CPPS and impaired semen parameters.

**Figure 2:**
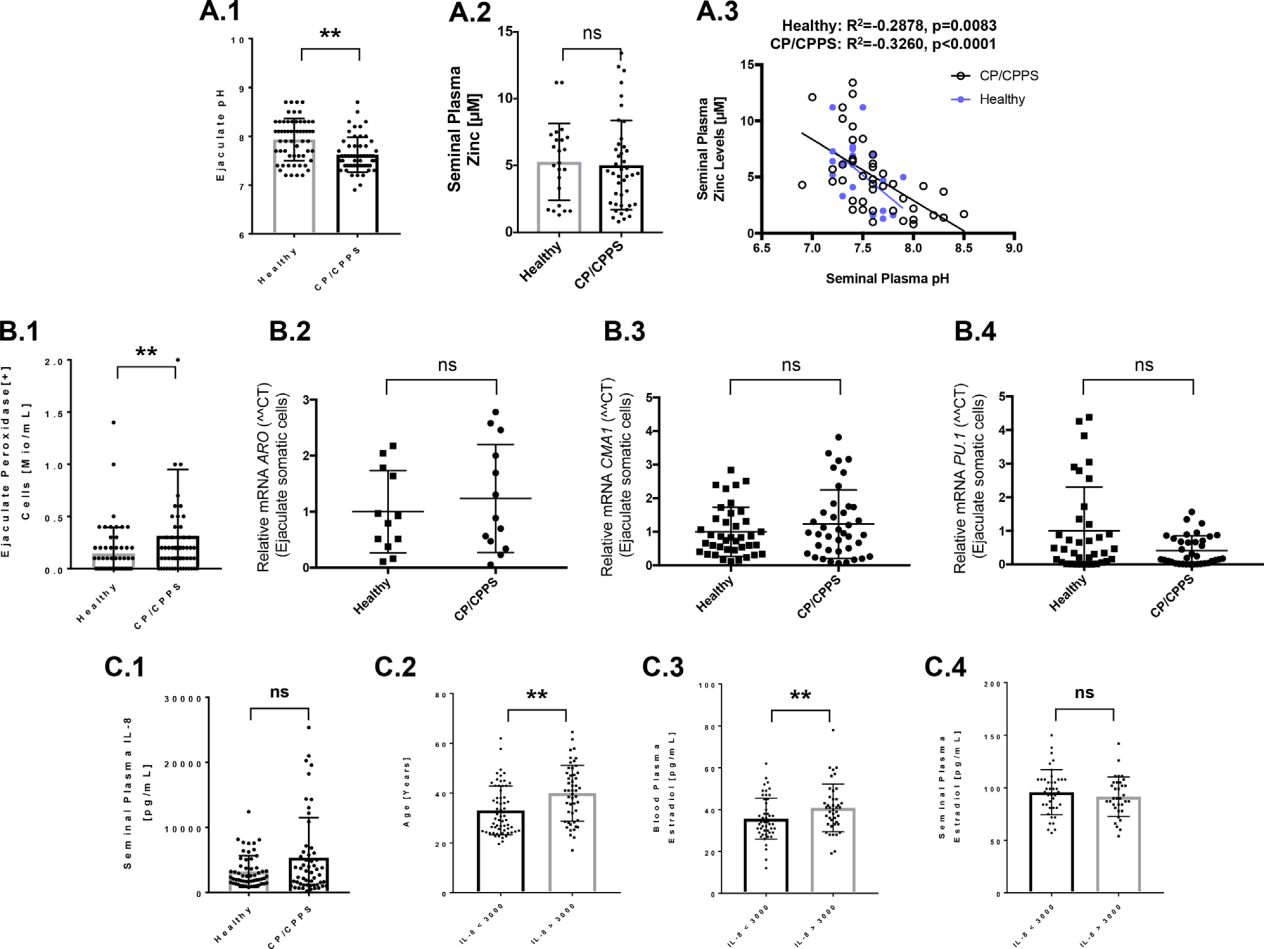
Analysis of semen parameters susceptible to changes under inflammatory conditions and expression of inflammation factors in ejaculated somatic cells (**A**) CP/CPPS patients’ semen possessed significantly reduced pH-values (**A.1**). Seminal plasma zinc levels were comparable between controls and patients (**A.2**). In both, controls and patients, semen zinc concentrations were highly significantly negative correlated to pH-values (**A.3**); (**B**) CP/CPPS patients’ semen possessed significantly increased number of peroxidase positive cells (leukocytes; represent the majority of somatic cells in ejaculates) (**B.1**). In ejaculated somatic cells cells, aromatase (*ARO*), mast cell chymase (*CMA1*) and *PU.1* (transcription factor of lymphoid and myeloid hematopoietic cells) mRNAs levels were not significantly different between CP/CPPS and controls (**B.2, B.3** and **B.4**); (**C**) Interleukin 8 (IL-8) levels did not differ significantly between controls and patients, although a trend for increased IL-8 was visible in CP/CPPS (**C.1**). Probands possessing very high IL-8 levels in semen (at cut-off of 3,000 pg/ml) were significantly older (**C.2**) and exhibited significantly increased E_2_ levels in blood (**C.3**), but not in seminal plasma (**C.4**). Abbreviations: ^**^p ≤ 0.01; ns: not significant (*p >* 0.05).

Seminal zinc, which is important for sperm motility amongst other functions, is mainly derived from prostatic secretions. The prostate fluid is known to be rich in citric acid, and the solubility of zinc increases with an increasing acidity. As CP/CPPS patients’ seminal plasma exhibited significantly decreased (more acidic) pH-values in comparison to controls (Figure 2A.1), we analyzed zinc levels in seminal plasma and its correlation to pH-values. Zinc concentrations per se were not changed in CP/CPPS in comparison to controls (Figure 2A.2). However, a significant negative correlation between zinc and pH-values could be observed in seminal plasma of CP/CPPS patients (R^2^ = –0.326, *p* < 0.0001) as well as controls (R^2^ = –0.2878, *p* = 0.0083) (Figure 2A.3).

Human semen contains besides spermatozoa and immature germ cells also a variety of somatic cells, including epithelial cells from the male tract and leukocytes. Our previous study demonstrated that ejaculated somatic cells from CP/CPPS patients exhibited epigenetic aberrations in *CXCR4* gene, a chemokine receptor often dysregulated in human tumors [25]. In order to get a more detailed profile of the white blood cells in ejaculates, we performed the peroxidase-positive leukocyte test and, additionally, an analysis of the expression of genes typical for inflammatory cells. We found that the number of peroxidase positive leukocytes was significantly increased in patients (Figure 2B.1). Somatic cells from the ejaculates of both CP/CPPS patients and healthy controls exhibited expression of aromatase (*ARO,* also estrogen synthase, is expressed in white blood cells), chymase (*CMA1*, mast cell marker) and *PU.1* (transcription factor of lymphoid and myeloid hematopoietic cells) at different levels (Figure 2B.2, 2B.3 and 2B.4). *ARO* and *CMA1* gene expression levels were comparable in CP/CPPS patients and controls (*p* > 0.05) (Figure 2B.2 and 2B.3), whereas *PU.1*, although also not significantly different, showed a trend towards lower expression in patients (Figure 2B.4). The concentration of the chemokine interleukin-8 (IL-8) was also measured in seminal plasma samples to evaluate the relationship of CP/CPPS to inflammation. While not significant, a trend for increased IL-8 levels was detected in CP/CPPS patients compared to controls (*p* > 0.05) (Figure 2C.1). However, by stratifying all studied men into low (<3,000 pg/ml) or high (≥3,000 pg/ml) seminal plasma IL-8 concentrations, we found that older men possessed significantly higher IL-8 concentrations (Figure 2C.2). High IL-8 levels (≥3,000 pg/ml) in seminal plasma were also significantly associated with high E_2_ levels in blood, but not in seminal plasma (Figure 2C.3 and 2C.4).

Our data show that ejaculates from both CP/CPPS patients and controls contain a considerable number of somatic cells expressing genes characteristic only for inflammatory i.e. white blood cells (leukocytes). In addition to impaired semen parameters, which have been previously associated with CP/CPPS, the somatic cells and leukocytes isolated from ejaculates may serve as a novel source of molecular information about the inflammatory state in the prostate of these patients. We also show that IL-8-concentrations in seminal plasma increase with age, independent from CP/CPPS diagnosis. As such, IL-8 would be an unreliable parameter to assess the extent of inflammation in CP/CPPS patients.

### Local epigenetic down-regulation of estrogen receptors *ESR1* and *ESR2* is associated with elevated estrogen levels in seminal plasma of CP/CPPS patients

**Figure 3:**
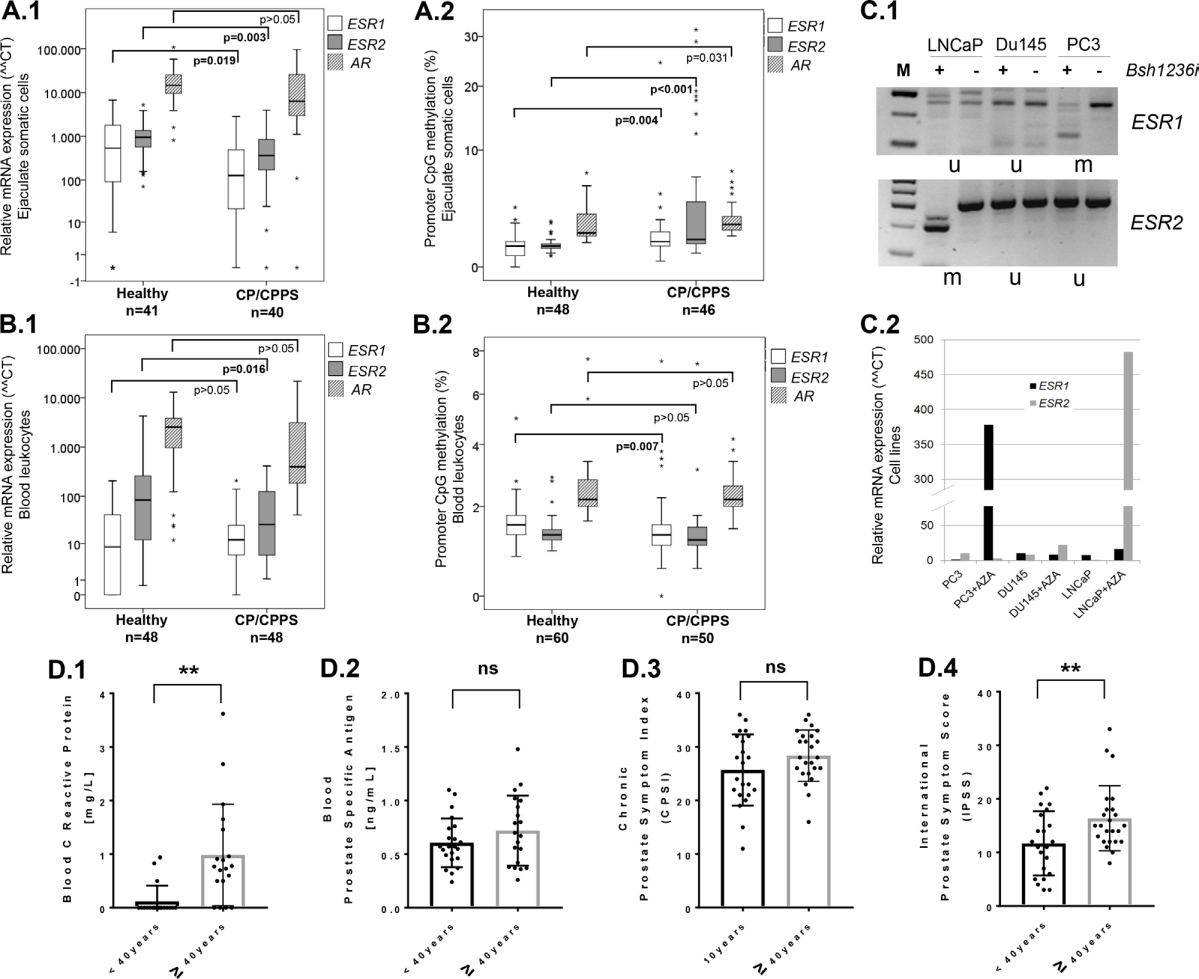
Epigenetic down-regulation of estrogen receptors *ESR1* and *ESR2* in CP/CPPS and deterioration of CP/CPPS symptoms in patients with advanced age (**A**) A significant decrease of expression of both estrogen receptor genes (*ESR1* and *ESR2*) was detected in somatic cells isolated from ejaculates of CP/CPPS patients in comparison to controls (**A.1**). By analyzing the promoter methylation of *ESR1* and *ESR2* in ejaculated somatic cells, a highly significant increase of cells having methylated *ESR1* and *ESR2* promoters was found in the CP/CPPS group (**A.2**). In contrast, androgen receptor (*AR*) gene expression and promoter methylation did not differ much between controls and patients (**A.1** and **A.1**); (**B**) Leukocytes isolated from blood of CP/CPPS and controls did not differ in gene expression of *ESR1* (**B.1**). CP/CPPS patients exhibited a reduced *ESR2* and *AR* expression, whereby for *ESR2* the difference was statistically significant (B.1). Methylation data showed a significant decrease of cells with methylated *ESR1* promoter in CP/CPPS patients’ blood leukocytes, whereas *ESR2* and *AR* promoters were comparable between patients and controls (**B.2**); (**C**) Prostate cancer cell lines LNCaP, DU145 and PC3 were analyzed by COBRA in *ESR1* and *ESR2* promoters. PC3 cells exhibited methylation (m) in *ESR1* promoter (Bsh1236i (CGCG)-specific restriction bands visible under “+”), whereas the other two were unmethylated (u) (**C.1**, upper gel picture). *ESR2* promoter was methylated in LNCaP cells and unmethylated in DU145 and PC3 (C.1, bottom gel picture). Treatment with 5-aza-2′-deoxycytidine (+AZA) led to re-expression of *ESR1* in PC3 cells and of *ESR2* in LNCaP cells (**C.2**); (**C**) Older CP/CPPS patients (≥40 years) exhibited extremely increased levels of CRP (C reactive protein) (**D.1**) and significantly higher IPSS (international prostate symptom score) than younger patients (<40 years) (**D.4**). The CPSI (chronic prostatitis symptom index), however, did not differ between younger and older patients (**D.3**). Prostate specific antigen (PSA) was also not significantly different in the blood of older and younger patients (**D.2**). Abbreviations: ^**^p ≤ 0.01; ns: not significant (*p >* 0.05).

Significantly elevated levels of E_2_ were found in seminal plasma of CP/CPPS patients and were positively correlated to urinary tract symptoms (Figure 1B.2 and 1B.3). In order to determine potential molecular reasons resulting in the local E_2_-excess in CP/CPPS patients, we analyzed somatic cells from ejaculates for gene expression and promoter CpG-methylation of the estrogen receptors, *ESR1* and *ESR2*, as well as the androgen receptor (*AR*). Hereafter, median values and ranges (minimum to maximum) for relative gene expression (delta-delta ct value) and promoter methylation (percentage of cells fully methylated in analyzed CpGs in *ESR1*, *ESR2* and *AR* promoters) are given. Transcription of both *ESR1* and *ESR2* were significantly decreased in CP/CPPS patients (*ESR1*: 125.9 (range 0 to 2,846.6); *ESR2*: 379.7 (range 0 to 4,933.3)) in comparison to healthy controls (*ESR1*: 537.5 (range 0 to 6,746.3); *ESR2*: 955.4 (range 70.3 to 5,226); *p* = 0.019 and *p* = 0.003, respectively; Mann–Whitney *U* test), whereas *AR* gene expression was not significantly changed (CP/CPPS: 7,454.2 (range 0 to 95,953); controls: 14,698 (range 815.6 to 10,8074); *p* > 0.05; Mann–Whitney *U* test) (Figure 3A.1). Methylation analyses revealed that *ESR1* and *ESR2* promoters were both significantly more methylated in ejaculated somatic cells of CP/CPPS patients (*ESR1*: 1.25% (range 0.25 to 24.5%); *ESR2*: 2% (range 0.5 to 31.5%)) than in controls (*ESR1*: 1% (range 0 to 5.25%); *ESR2*: 1% (range 0.5 to 6.13%); *p* = 0.004 and *p* < 0.001, respectively; Mann–Whitney *U* test), whereas the methylation status of *AR* was only slightly changed (CP/CPPS: 2.4% (range 1.6 to 7.4%); controls: 1.8% (range 1.2 to 7.2%); *p* = 0.031; Mann–Whitney *U* test) (Figure 3A.2). Particularly notable was a remarkable high methylation degree of *ESR1* and *ESR2* promoters (up to 20–30%) in a number of CP/CPPS cases, whereas the controls were inconspicuous (Figure 3A.2).

Leukocytes isolated from blood did not differ significantly (*p* > 0.05) between CP/CPPS patients and controls with regard to *ESR1* and *AR* gene expression (CP/CPPS: 11 (range 0 to 206.7) and 455 (range 40.8 to 21,718); controls: 9.4 (range 0 to 260.5) and 2,378 (range 11.9 to 12,988), respectively) (Figure 3B.1). However, CP/CPPS patients exhibited a significantly decreased *ESR2* expression (25.9 (range 1.1 to 413)) in comparison to controls (74.7 (range 0.6 to 4,284); *p* = 0.016, Mann–Whitney *U* test) (Figure 3B.1). Methylation analyses revealed a significant difference in *ESR1* promoter (CP/CPPS: 1.25% (range 0 to 7.5%); controls: 1.5% (range 0.75 to 5%); *p* = 0.007, Mann–Whitney *U* test), but not in *ESR2* (CP/CPPS: 1.1% (range 0.5 to 7.4%); controls: 1.3% (range 0.9 to 2.9%); *p* > 0.05) and *AR* promoter (CP/CPPS: 2.2% (range 1.4 to 4.2%); controls: 2.2% (range 1.6 to 7.6%); *p* > 0.05) (Figure 3B.2).

In order to show the effect of *ESR1* and *ESR2* promoter methylation and demethylation on gene expression, prostate cancer cell lines LNCaP, DU145 and PC3 were treated with 5-aza-2′-deoxycytidine (AZA). AZA is a cytosine analog that inhibits the DNA methyltransferase activity and induces demethylation and reactivation of silenced genes. In PC3 cells, which possessed a hypermethylated *ESR1* promoter (Figure 3C.1) and had no *ESR1* expression, AZA treatment led to re-expression of *ESR1* (Figure 3C.2). In the same way, in LNCaP cells possessing a hypermethylated *ESR2* promoter (Figure 3C.1) and gene silencing, AZA treatment led to re-expression of *ESR2* (Figure 3C.2). These results confirm that the expression of *ESR1* and *ESR2* genes is influenced by promoter methylation.

So, our findings in ejaculated somatic cells showed that a local (in male genital compartment) epigenetic down-regulation of estrogen receptor genes *ESR1* and *ESR2* was characteristic for CP/CPPS patients and associated with local estrogen excess in these patients. These data suggest that *ESR1* and *ESR2* methylation and expression status in ejaculated somatic cells may be suitable molecular markers for CP/CPPS. In contrast, the *AR* was not changed and hence would be less suitable. From our analyses in blood we could not obtain a coherent picture: CP/CPPS patients showed a lower transcription level of *ESR2* and, however, a decreased *ESR1* methylation compared to healthy controls.

### Deterioration of CP/CPPS symptoms and elevated C-reactive protein in patients with advanced age

In order to evaluate, whether age and the associated hormonal changes that occur upon aging, affect the disease severity and symptoms of CP/CPPS, we stratified patients into two age-groups (separated using the median cohort age; <40 years *versus* ≥40 years) and analyzed with regard to C-reactive protein (CRP, a marker of inflammation), prostate specific antigen (PSA) and disease severity based on CPSI and IPSS (international prostate symptom score) questionnaires. CRP was significantly affected, with older patients having significantly elevated levels of CRP in blood (*p ≤* 0.01) (Figure 3D.1). PSA levels and the CPSI score were not significantly different in older patients compared to younger ones (*p* > 0.05) (Figure 3D.2 and 3D.3). However, the IPSS questionnaire score was also significantly increased in older CP/CPPS patients (*p ≤* 0.01; Figure 3D.4).

### Impact of mast cells in chronic inflammation of the prostate

**Figure 4:**
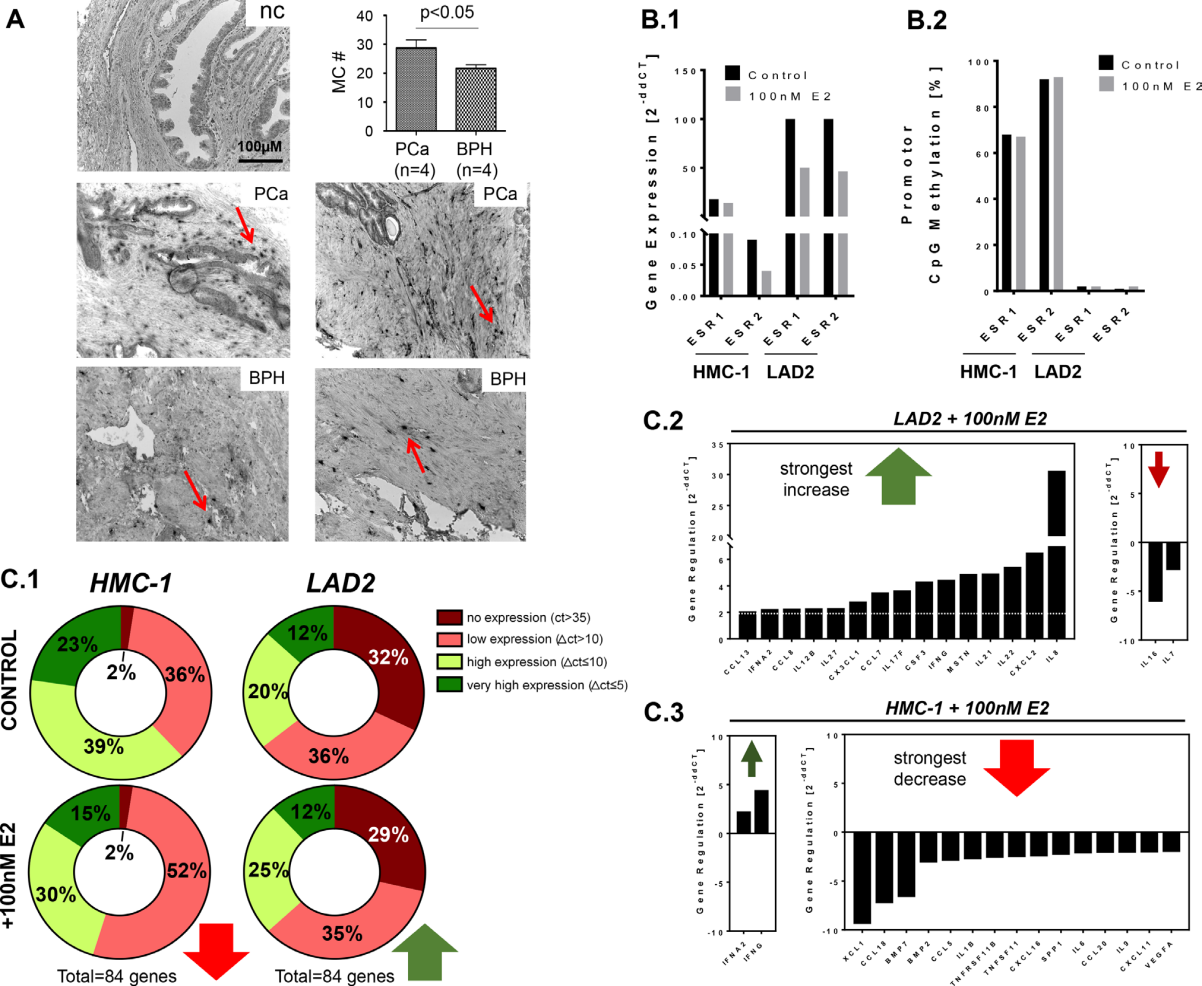
Incidence of mast cells in the prostate, and different effects of estradiol excess on inflammatory gene expression in mast cells (**A**) Tryptase (mast cell specific protease)-positive foci (marked by arrows) were found to be abundantly present in tumor-associated stroma of prostate carcinoma (PCa) tissues. Benign prostate hyperplasia (BPH) samples exhibited fewer mast cells (MC #) as it is shown in the diagram (A; representative examples for PCa and BPH are shown); (**B**) Two human mast cell lines, HMC-1 and LAD2, were analyzed with regard to gene expression and CpG-promoter methylation of *ESR1* and *ESR2* before and after short term (24 h) stimulation with 100 nM estradiol (E2). Untreated HMC-1 cells possessed low *ESR1* and *ESR2* expression (B.1) and hypermethylated *ESR1* and *ESR2* promoters (60–90%) (**B.2**). In contrast, untreated LAD2 cells possessed high *ESR1* and *ESR2* expression (**B.1**) and unmethylated *ESR1* and *ESR2* promoters (1–2%) (**B.2**). Expression levels of *ESR1* and *ESR2* after E2-treatment were 2-fold reduced in LAD2, and *ESR2* was 2-fold reduced in HMC-1 (**B.1**). Short term stimulation with E2 did not change the methylation degrees of *ESR1* and *ESR2* promoters in both cell lines; (**C**) In order to reveal the effect of estradiol excess on transcription profiles of HMC-1 and LAD2 cells, a transcription profiler for 84 inflammatory genes was utilized. Untreated LAD2 and HMC-1 cells possessed very different expression profiles (**C.1**, Control). E2 stimulation led to diverging reactions in HMC-1, the majority of responsive genes were down-regulated (increase of the percentage of low expressed genes, in red), whereas LAD2 the majority of responsive genes were up-regulated (increase of the percentage of high expressed genes, in green) (**C.1**, +100 nM E2). Genes reacting with at least 2-fold gain or loss of expression upon E_2_ stimulation (green and red arrows, respectively) are shown for LAD2 and HMC-1 (**C.2** and **C.3**, see also Supplementary Figures 2 and 3).

Mast cells are a type of leukocytes with myeloid origin, which are essential in mediating inflammatory processes, regulating a variety of both adaptive and innate immune responses [26]. Prostate tumors, which are frequently accompanied by prostatic inflammation, contain significantly elevated numbers of mast cells at the tumor interface, with the tumor stroma promoting increased mast cell recruitment and activation [16]. Consistent with this and other previous studies, we found the presence of mast cells in benign prostate hyperplasia (BPH) and prostate carcinoma (PCa) tissue samples, demonstrated by the presence and release of the mast specific protease, tryptase (Figure 4A). PCa samples (*n* = 4) showed a slightly increased number of mast cells in comparison to BPH (*n* = 4) (PCa: 28.7 ± 2.8 versus BPH: 21.7 ± 1.2, *p* < 0.05, *t*-test) (Figure 4A).

### Mast cell lines HMC-1 and LAD2 differ in expression and promoter methylation of *ESR1* and *ESR2* genes

In prostate tumors it has also been demonstrated that mast cell recruitment and activation is induced by estradiol (E_2_) [16]. Our current results revealing increased E_2_ levels in the seminal plasma of CP/CPPS patients (Figure 1B.2), along with the expression of mast cell chymase in the ejaculated somatic cells of patients and controls (Figure 2B.3), suggest an involvement of mast cells in CP/CPPS. In order to evaluate the effect of increased E_2_ on mast cell function we analyzed two human mast cell lines, HMC-1 (progenitor-like) and LAD2 (mature), before and after treatment with high dose (100 nM) E_2_ for 24 hours. Both lines express the two estrogen receptors, and our initial analyses showed that in untreated HMC-1 cells, *ESR1* and *ESR2* promoters were hypermethylated (68% and 92%, respectively) and gene transcriptions were accordingly low (Figure 4B.1 and 4B.2). These data from the HMC-1 cells are reminiscent of what is detectable in ejaculated somatic cells from CP/CPPS patients, where significantly lower expression of *ESR1* and *ESR2* and increased promoter methylation is observed in comparison to healthy controls (Figure 3A.1 and 3A.2). In contrast, in untreated LAD2 cells, *ESR1* and *ESR2* genes were transcribed at high levels and the promoters were almost unmethylated (2% and 1%) (Figure 4B.1 and 4B.2). The short term (24 h) E_2_ treatment led to a halving of *ESR1* and *ESR2* gene expression in LAD2, and of *ESR2* in HMC-1 cells (Figure 4B.1). No changes were found after 24 h E_2_ treatment with regard to *ESR1* and *ESR2* promoter methylation in HMC-1 as well as in LAD2 cells (Figure 4B.2).

### Increased estrogen levels lead to suppression of cytokines and chemokines in HMC-1 and their activation in LAD2

The expression of a panel of 84 cytokines and chemokines was examined in untreated and E_2_-stimulated HMC-1 and LAD2 cells using the RT^2^ Profiler PCR array “Human cytokines and chemokines” (Qiagen). In untreated HMC-1 cells, the majority of genes (52 out of 84, 62%) were expressed at high levels (delta Ct ≤ 10 in qPCR), whereas in untreated LAD2, only 27 out of 84 genes (32%) had a high expression and the majority (68%) had low expression (delta Ct > 10) (Figure 4C.1). Stimulation with E_2_ had opposite effects in these two lines (Figure 4C.2 and 4C.3). While in HMC-1 cells the majority of E_2_-responsive genes was transcriptionally suppressed (21 out of 23 responsive genes were at least 2-fold down-regulated), a number of genes in LAD2 cells showed increased expression (15 out of 18 responsive genes were at least 2-fold up-regulated). Among the E_2_-suppressed genes in HMC-1 cells, *XCL1, CCL18*, *CXCL2* and *BMP7* showed the strongest (6- to 9-fold) down-regulation (Figure 4C.3 and Supplementary Figure 2). Among E_2_-activated genes in LAD2 cells, *IL-8*, *CXCL2*, *IL22*, *IL21*, *MSTN, IFNG* and *CSF3* showed the strongest (4- to 30-fold) up-regulation (Figure 4C.2 and Supplementary Figure 2).

Altogether, different promoter methylation states and herewith associated basal activities of *ESR1* and *ESR2* in HMC-1 (progenitor-like) and LAD2 cells (mature) associated with very different expression profiles of cytokines and chemokines before and after E_2_ stimulation. Specifically, in untreated HMC-1 cells, the majority of analyzed inflammatory genes was already actively transcribed, while, in contrast, in control LAD2 cells the majority of genes was silenced or transcribed at low levels. Treatment with E_2_ led in HMC-1 mainly to immune-suppression, whereas LAD2 cells reacted with activation of immune factors.

### The methylation profile of HMC-1 and LAD2 cells is similar and does not change under short term estrogen stimulation

The methylation profile of 24 genes with well documented roles in cytokine biosynthesis was evaluated in untreated and E_2_-stimulated (100 nM, 24 hours) mast cells using the EpiTect Methyl II Signature PCR array “Human cytokine production” (Qiagen) and pyrosequencing (Supplementary Figure 3). LAD2 and HMC-1 cells were both found to be hypermethylated in the promoters of *ELANE*, *FOXP3*, *INHA*, *INHBA*, *SMAD3*, *CXCL12* and *IL-13* (17% to 100%) (Supplementary Figure 3). Additionally, LAD2 cells were also hypermethylated in the *STAT5A* promoter, and HMC-1 cells in *IGF2BP2*, *ESR1*, *ESR2* and *AR* promoters (Supplementary Figure 3). All other genes analyzed were unmethylated. E_2_ stimulation did not change the methylation levels of the genes examined, except in *BCL3* (0% to 61%) and *TLR2* (0% to 56%) in HMC-1 cells, and *GATA3* (51% to 0%) and *INHA* (66% to 28%) in LAD2 cells.

## DISCUSSION

CP/CPPS is a disease of all ages and patients are characterized by their symptoms. In a large international study comprising 1,563 CP/CPPS patients, the mean age was 47.2 years (SD - 15.7 years) [27]. In this study the included patients were somewhat younger, but generally matched other published cohorts. Decreasing testosterone levels with age [28] represent a possible confounding factor in a study with a wide age range as ours. This fact has been addressed by comparing with an age matched healthy control population.

The present results demonstrate that estradiol (E_2_) excess in seminal plasma is a characteristic of CP/CPPS patients and correlates significantly with the NIH-CPSI urinary tract symptom subscore. The increase in local estrogen levels could be explained by elevated aromatase expression within the reproductive tissues of CP/CPPS patients as this was not apparent in somatic cells isolated from ejaculates. Interestingly, in addition to locally elevated E_2_ levels, we found in ejaculated somatic cells of CP/CPPS patients a significant down-regulation of *ESR1* and *ESR2* gene expression, which associated with a significant higher methylation of *ESR1* and *ESR2* promoters. It remains unclear, whether these epigenetic changes affect the leukocytes in ejaculates, which represent the majority of somatic cells in semen, or other somatic cells (e.g. epithelial cells from urogenital tract), which can also be present even if in low numbers. Further studies should address and clarify this issue. However, we could detect a significant higher number of peroxidase-positive cells (leukocytes) in CP/CPPS patients’ ejaculates than in controls. Moreover, in CP/CPPS patients, but not in controls, a significant correlation between estradiol levels in seminal plasma and in blood was detected, indicating a CP/CPPS specific systemic and local deregulation of E_2._ These novel findings in CP/CPPS patients demonstrate that estrogen and estrogen receptors’ deregulation may play an important role in the chronification of prostatitis and, together with our previous results [25], suggest that semen is a suitable source for development of diagnostic biomarkers for CP/CPPS.

In contrast to semen, there were no significant differences detectable with regard to systemic (in blood) estradiol levels between CP/CPPS and controls. We found that regardless of the presence of CP/CPPS, men exhibiting high IL-8 levels in seminal plasma (<3,000 pg/ml vs. <3,000 pg/ml) were on average significantly older and had significantly increased blood estradiol concentrations. Thus, IL-8 seems to be an age-dependent factor and must be used in the clinical practice with caution. By analyzing leukocytes isolated from blood, we found a significantly decreased methylation in *ESR1* promoter in CP/CPPS, but we did not detect any changes in *ESR1* gene expression. Furthermore, blood leukocytes from CP/CPPS patients possessed a significantly decreased *ESR2* expression, whereas *ESR2* promoter did not differ from controls with regard to the methylation status. At this point we cannot deliver a conclusive explanation about the deregulation of *ESR1* and *ESR2* gene activity in peripheral blood of CP/CPPS patients, and further differential studies on different types of white blood cells are needed. It is known that ESR1 (or ERα) is expressed in circulating peripheral macrophage- and dendritic cell precursors (MDPs) and plays a critical role in dendritic cell (DC) maturation [29]. In a mouse model it was shown that macrophages, a subtype of DCs, had significantly increased TNFα and IL-1B levels and that this effect was ERα dependent [30]. The potential of estrogenic imbalance to skew dendritic cell maturation was shown in postmenopausal women. Plasmocytoid dendritic cells of postmenopausal women showed reduced toll like receptor 7-mediated response, compared to a younger cohort, and this impairment was partially reversed by estradiol supplementation [31]. DCs develop from circulating monocytes and mediate adaptive immunity by antigen-presentation to T-cells, which have been connected to autoimmunity in CP/CPPS. Prostate antigen immunization of wild type mice (C57BL/6) resulted in an IL-4/IL-12 dependent infiltration of CD4+ T-cells and macrophages [13], positing a role for CD4+ T-cells in prostatic inflammation. An earlier case-controlled study also showed that peripheral CD4+ T-cells isolated from peripheral blood buffy-coats of CP/CPPS patients had an increased reactivity to prostatic alkaline phosphatase and PSA [32]. Thus, the systemic deregulation of *ESR1* and *ESR2* genes found in CP/CPPS could indicate a dendritic cell mediated autoimmunity for CP/CPPS. If the observed systemic changes are causative for CP/CPPS or are merely a response to local prostate inflammation remains to be determined.

The prostate has the highest zinc concentration of all organs in the body [33] and semen zinc levels are correlated with fertility parameters including alpha-glycosidase, a function marker of epididymis [34]. Semen zinc is mainly derived from prostatic secretions. The prostate fluid is known to be rich in citric acid, and the solubility of zinc increases with an increasing acidity. Accordingly, in semen of CP/CPPS patients as well as controls we found a significant negative correlation between zinc concentrations and pH-values. In a previous study, zinc levels were also found to be reduced in ejaculates of CP/CPPS patients [35]. However, in our study cohorts we could not detect a difference in semen zinc concentrations between CP/CPPS patients and controls. This may be due to the fact that only data for older (pre-vasectomy) controls were available as reference. Nevertheless, zinc plays an important role for the immune system [36] and dietary zinc also has the potential to alter steroid hormone turnover and modulate testosterone aromatization [37]. Moreover, BPH and PCa tissues also have significantly reduced zinc levels compared to normal prostate tissue [38]. The potential of zinc deficiency to skew the inflammatory reaction is supported by experiments in pro-myeolid (HL-60) cells, which increased production of IL-1B and TNFα after zinc depletion [39]. Strikingly, systemic IL-1B and TNFα were significantly elevated and positively correlated with patient anxiety and depression scores in a large clinical study on CP/CPPS patients [40]. Furthermore, zinc supplementation to CP/CPPS patients led to a significant decrease of their NIH-CPSI score in the follow-up observation after 12 weeks [41].

Mast cells are resident in the human prostate, and have been implicated to play a role in CP/CPPS as well as prostate cancer [16, 42]. Significantly, they are also estrogen responsive [16]. In order to gain mechanistic insight regarding the elevated estrogen levels we observed in the seminal plasma of CP/CPPS patients and the effect on human mast cells, we used two human mast cell lines possessing different expression of the estrogen receptors (ERα and ERβ) and stimulated them with a high concentration of estradiol (100 nM). These cell lines, HMC-1 and LAD2, are extensively characterized and represent the gold-standard for studies of mast cell function *in vitro*. Similar to leukocytes in the ejaculates of CP/CPPS patients, untreated HMC-1 cells possessed epigenetically (through promoter methylation) caused low *ESR1* and *ESR2* gene expression. In contrast, *ESR1* and *ESR2* were both actively expressed and the promoters were unmethylated in untreated LAD2 cells. The cell lines also differ remarkably in their basal inflammatory gene expression profile and response to estradiol stimulation. In untreated HMC-1 cells 52 out of 84 analyzed inflammatory factors were actively transcribed, whereas in LAD2 cells only 27 out of 84 were expressed. Estradiol stimulation of HMC-1 cells caused a suppression of 21 inflammatory genes (out of 23 responsive genes in total), and in LAD2 cells, an activation of 15 genes (out of 18 responsive). HMC-1 and LAD2 cells also vary considerably in cell properties and culture conditions. LAD2 cells, phenotypically resembling more mature mast cells, grow much slower and are more difficult to culture, compared to HMC-1 cells, which are immature and more progenitor-like. Additionally, a comparison of both cell lines with mature skin mast cells (sMC) also reported that LAD2 cells are intermediately differentiated, while HMC-1 cells are very immature malignantly transformed mast cells [43].

Elevated estrogen levels and altered expression of the estrogen receptors, particularly an altered ERα:ERβ ratio, are also factors that are associated with prostate cancer. Previous reports have proposed that ESR1 and ESR2 could have a tumor suppressor role in prostate gland. For example, loss of *ESR1* transcription and ESR1 protein by promoter methylation, respectively, increased with progression of prostatic disease from BPH to low grade and to high grade cancer [44–46].Most reports on ESR2 expression concur, that levels decline in localized prostate cancer with increasing grade from prostatic intraepithelial neoplasia through low to high grade Gleason scores [47–50].The loss of ESR2 expression in organ confined prostate cancer has been shown to be epigenetically regulated by progressive hypermethylation of *ESR2* promoter [46, 51]. The capability of estrogen as a ligand to down-regulate ERα is well established [52, 53]. In the breast cancer MCF-7 cell line it has also been shown that the phytoestrogens quercetin and genistein share this capability [21], which makes the use of quercetin in treatment of CP/CPPS questionable or, at least emphasizes the need of an individualized therapy with an involvement of molecular analyses prior to treatment. Similar regulation of ERβ occurs, but can differ between tissue types. A study investigating the effect of estrogen on its receptors found estradiol-mediated ERβ down-regulation in MCF-7, but up-regulation in human aortic smooth muscle cells [54]. These and our current data support the growing body of evidence implicating chronic prostatitis in the development of prostate malignancy.

In summary, our results demonstrate that estradiol excess in seminal plasma is a characteristic of patients with CP/CPPS. This is also associated with a local epigenetic down-regulation of the estrogen receptors, which is traceable in the somatic cells of the ejaculate. Estrogen was also shown to alter inflammatory cytokine and chemokine expression in mast cells in opposite directions depending on the epigenetic status and transcriptional activity of estrogen receptors, which in turn are implicated in CP/CPPS and prostate cancer development. Overall, these novel findings suggest that estrogen action may play an important role in the chronification of prostatitis. Additionally, these data also suggest that somatic cells from the ejaculate could reflect the inflammation status of the prostate, and may be a viable resource to develop supportive molecular markers for CP/CPPS.

## MATERIALS AND METHODS

### Collection and analysis of liquid biopsies from CP/CPPS patients and healthy controls

Whole blood and semen samples were collected from CP/CPPS patients (*n* = 50, median age 39.76, range 23–65) and healthy men (controls) without any preexisting urological conditions (*n* = 61, median age 36.77, range 20–69) in the Clinic of Urology, Pediatric Urology and Andrology, JLU Giessen, Germany. The control group comprised volunteers (*n* = 40) and men requesting vasectomy (*n* = 21). The latter cohort was added in order to get an age-matched control cohort and to exclude age-associated effects on hormonal balance. From patients undergoing vasectomy, we used pre-vasectomy peripheral blood and ejaculates, respectively. All study participants gave their written informed consent and the study was approved by the Ethics Commission of the JLU Giessen (ethical vote, AZ.: 55/13).

The clinical presentation of CP/CPPS patients and healthy volunteers was confirmed according to guidelines [7, 55, 56], including the use of the German version of the Chronic Prostatitis Symptom Index from the National Institute of Health (NIHCPSI) [57] as we previously described [25]. Infection was excluded with microbiological cultures and gene amplification analysis [57, 58]. Since the classification to inflammatory (IIIa) and non-inflammatory (IIIb) CP/CPPS might present two different stages of the same condition [59], both sub-categories were included in our patient cohort.

Blood samples from CP/CPPS patients were routinely analyzed for total testosterone, C-reactive protein (CRP) and prostate specific antigen (PSA) levels in the central laboratory of our university hospital (ADIVA, Siemens Health Care, Erlangen, Germany). Estradiol concentrations in blood and seminal plasma of patients and controls were analyzed separately at the Institute of Laboratory Medicine and Pathobiochemistry, Molecular Diagnostics, UKGM Giessen. Semen parameters of patients and controls were routinely analyzed according to the WHO-2010 recommendations [60]. Seminal plasma was separated from the cellular fraction by centrifugation (5 minutes at 600 g) and analyzed for polymorpho-nuclear elastase, IL-8, alpha-glycosidase and zinc with standardized methods [25]. The cellular fractions (ejaculate pellets) were stored at –80°C and used further for isolation of leukocytes (refer below). CP/CPPS patients and healthy volunteers were examined and evaluated based on CPSI, IPSS and HADS questionnaires. All clinical and pathological data gathered are summarized in Supplementary Figure 1.

### Isolation of leukocytes from whole blood and ejaculate samples

Frozen (–80°C) EDTA blood samples (3 ml) were thawed on ice and incubated for 10 minutes with 5 volumes of red blood cell lysis buffer (15.5 mM NH_4_Cl, 1 mM KHCO_3_ and 0.01 mM tetra-sodium EDTA in di-ethyl-pyrocarbonate treated deionized water; pH 7.3). Cells were centrifuged (10 minutes at 600 g) and pellets were incubated with 1.5 ml fresh buffer on ice for another 10 minutes. Blood leukocytes were obtained by centrifugation (5 minutes at 600 g). Ejaculate leukocytes were isolated from frozen-stored ejaculate pellets by density gradient centrifugation. Briefly, ejaculate pellets were thawed on ice and dissolved in 600 μl DMEM-F12 medium (Gibco). Samples were then placed on top of a pre-layered Histopaque^®^1077 gradient (1.5 ml 90% + 1.5 ml 50% + 1.5 ml 20%; all solutions at 4°C) in 12ml-polypropylene-tubes (Sarstedt) and centrifuged (30 minutes at 400 g). The phenol-red from DMEM-F12 allowed for convenient identification of resulting phases. Upper fraction contained leukocytes, and the pellet contained sperm cells. Leukocytes were pelletized by centrifugation for 5 minutes at 600 g and 4°C. Leukocytes from blood and ejaculates were washed twice with PBS and frozen at –80°C until DNA and RNA isolations.

### DNA and RNA extractions from leukocytes

Blood and ejaculate leukocyte samples were thawed on ice and dissolved in 1ml peqGOLD TriFast™ reagent (Peqlab). The aqueous phase was used for RNA isolation and DNA was isolated from the organic phase. RNA and DNA isolations were performed according to the manufacturer's protocol (Peqlab). RNA was digested with 1 unit DNAseI in 20 μl volume following the manufacturer's (NEB) instructions, purified and dissolved in 20 μl of DEPC treated nuclease-free water. DNA was recovered from the same sample by addition of 0.5 ml Back Extraction Buffer (4 M guanidine thiocyanate, 50 mM sodium citrate and 1 M Tris) to the organic phase. RNA and DNA concentrations were measured on NanoDrop 1000 (Thermo Scientific) and stored at –80°C until use.

### 5-aza-2′-deoxycytidine treatment of prostate cancer cell lines and COBRA analysis

Human prostate cancer cell lines PC3, LNCaP and DU145 were used in order to analyze the effect of *ESR1* and *ESR2* promoter methylation and demethylation, respectively, on gene expression. As described in our previous study [25], 30% confluent cell plates were treated with 5 μM 5-aza-2′-deoxycytidine (Sigma Aldrich) for 72 hours. On day 4, the cells were harvested and the nucleic acids were isolated and subjected for *ESR1* and *ESR2* expression analyses (by RT-qPCR as described below) and promoter methylation analyses by COBRA (combined bisulfite restriction analysis; Table 1) using the CGCG-specific enzyme *Bsh1236i* (Thermo Fisher Scientific).

**Table 1:** Primer sets used for pyrosequencing, COBRA and RT-qPCR

Method/Gene	Upper (5′-3′)	Lower (5′-3′)	Ta	PCR size
**Pyrosequencing/*ESR1***	Hs_ESR1_01_PM PyroMark CpG assay (Qiagen, 4 CpGs)		60	114bp
**Pyrosequencing/*ESR2***	Hs_ESR2_01_PM PyroMark CpG assay (Qiagen, 8 CpGs)		60	192bp
**Pyrosequencing/*AR***	Hs_AR_01_PM PyroMark CpG assay (Qiagen, 5 CpGs)		60	167bp
**COBRA/*ESR1***	AGYGTGTTTTYGAGT TYGTT	ACTCCCRCAACTCCCTAAAC	60	247bp
**COBRA/*ESR2***	TGTGGGTGGATTAGG AGTYG	RCCTACTCTTCRCCCTACAA	60	299bp
**RT-qPCR/*ESR1***	TGCAGGGAGAGGAGTTTGTG	GGACAGAAATGTGTACA CTCCAGA	60	71bp
**RT-qPCR/*ESR2***	GATGAGGGGAAATGCGTAGA	GATCATGGCCTTGACACAGA	60	120bp
**RT-qPCR/*AR***	GCAGGAAGCAGTATCCGAAG	GACACCGACACTGCCTTACA	60	144bp
**RT-qPCR/*CMA1***	GTCCCACCTGGGAGAATGTG	TCTTGCAGAGTGTCTGAGCC	60	80bp
**RT-qPCR/*ARO***	AGGAGGTGACCAATGAATCG	CACGATAGCACTTTCGTCCA	60	116bp
**RT-qPCR/*PU.1***	GTGCCCTATGACACGGATCT	CCAGTAATGGTCGCTATGGCT	60	93bp
**RT-qPCR/*TUBA1B***	CGTGCCCCGGGCTGTGTTT	GCAGCATCTTCCTTGCCTGTGA	60	117bp

**Abbreviations:** COBRA: combined bisulfite restricition analysis (Y = C + T; R = A + G); RT-qPCR: reverse transcription followed by quantitative real-time PCR; Ta: annealing temperature; PCR size: length of PCR product; *ESR1*: Estrogen Receptor Alpha; *ESR2*: Estrogen Receptor Beta; *AR*: Androgen Receptor; *CMA1*: Human mast cell chymase 1; *ARO*: Aromatase; *PU.1*: hematopoietic transcription factor SPI1 binding to PU-box; *TUBA1B*: tubulin alpha 1b.

### Estrogen stimulation of HMC-1 and LAD2 cells, and RNA and DNA isolations

Human HMC-1 mast cells (kindly provided by Dr J. Butterfield; Mayo Clinic, USA) were cultured as previously described [16]. LAD2 cells (kindly provided by Dr A. Kirshenbaum; National Institutes of Health, USA) were maintained as previously described [61]. Cells were then seeded into 10cm dishes at densities of ∼0.176 × 10^6^/cm^2^ (HMC1, 1 × 10^7^/dish) and ∼0.529 × 10^5^/cm^2^ (LAD2, 3 × 10^6^/dish). Treatments (100 nM estradiol or ethanol vehicle control) were done twice (for DNA and RNA, respectively) under the same conditions. After 24 hours, cells were washed with PBS (5 minutes centrifugation at 400 g) and resuspended in 50 μL PBS for DNA isolation or 100 μl RNA-later (Sigma) for RNA isolation. For DNA extraction, cell pellets were digested for 1 hour with 0.75 μg/μL proteinase K (Carl Roth) at 56°C. 500 μL of Phenol/Chloroform/isoamylalcohol (25:24:1) were added, and phase separation was acquired by centrifugation (10 minutes at 13.000 rpm). DNA samples were precipitated with 1 volume isopropanol, 1/10 volume sodium acetate (3 M) and 5 μg glycogen. RNA from cell pellets was isolated with peqGOLD TriFast™ reagent (Peqlab) according to manufacturer's protocol. RNA and DNA concentrations were measured on the NanoDrop-1000 (Thermo Scientific) and stored at –80°C until use.

### Gene expression analyses using RT-qPCR and RT^2^ Profiler

Leukocytes isolated from blood and ejaculates of CP/CPPS patients and healthy controls were analyzed for relative gene expression of *ESR1*, *ESR2*, *AR*, *CMA1*, *ARO* and *PU.1* by RT-qPCR (reverse transcription followed by quantitative real-time PCR) using the CFX96 Touch™ RealTime PCR Detection System (Biorad) and RotorGene SYBR Green PCR Kit (Qiagen) (Table 1). The house keeping gene *TUBA1B* (alpha-tubulin) was used as reference gene (Table 1). An RNA sample isolated from blood leukocytes of a healthy donor was used as internal standard sample (calibrator). RNA samples (each 2 μg) were reverse transcribed in cDNA with MMLV-RT (Promega) according to manufacturer's protocol. The cDNA samples were purified with the Nucleotide Removal Kit (Qiagen). Quantitative PCRs were performed in duplicates. Relative expression levels were calculated with the ΔΔC_T_ method.

Untreated and E_2_-stimulated mast cell lines HMC-1 and LAD2 were analyzed with regard to relative gene expression of 84 inflammatory factors using the RT^2^ Profiler™ “Human Cytokines and Chemokines PCR Array” (Qiagen) (E_2_-responsive genes are shown in Supplementary Figure 2). Briefly, RNA samples (each 2 μg) were reverse-transcribed with the RT^2^ First Strand Kit (Qiagen). The cDNA samples were purified with the Nucleotide Removal Kit (Qiagen) and applied for the RT^2^ Profiler™. Relative gene expression levels were calculated according to manufacturer's suggestion by using the SA Biosciences PCR Array Data Analysis Web portal (Qiagen). Five housekeeping genes (*ACTB*, *B2M*, *GAPDH*, *HPRT1* and *RPLP0*) were included. The genes *B2M*, *GAPDH* and *HPRT1* showing the most stable results, were chosen as reference genes for normalization of values generated by RT^2^ Profiler™. RT-qPCRs for *ESR1*, *ESR2*, *AR* and *TUB1A* were done for HMC-1 and LAD2 RNA samples in triplicates as described before.

### CpG-promotor methylation analyses by pyrosequencing and EpiTect Methyl II Profiler

Leukocytes isolated from blood and ejaculates of CP/CPPS patients and healthy controls, and mast cell lines HMC-1 and LAD2 (untreated and E2-stimulated) were analyzed for CpG-promoter methylation of *ESR1*, *ESR2* and *AR* genes. Therefore, 1 μg DNA was bisulfite-treated using the EZ DNA Methylation™ Kit (Zymogen). Bisulfite-treated DNA samples were subsequently amplified and pyrosequenced using appropriate primer sets (Table 1, Qiagen) and the PyroMark Q24 System (Qiagen). Prior pyrosequencing the PCR products were controlled on an agarose gel (2%). Methylation values were analyzed using PyroMark Q24 Software (Qiagen; quantitative analysis of 4 CpG sites in *ESR1*, 8 CpGs in *ESR2* and 5 CpGs in *AR*).

Both mast cell lines were analyzed for CpG-promoter methylation of 22 inflammatory factors using the EpiTect Methyl II “Human Cytokine Production Signature Panel” (Qiagen) (Supplementary Figure 3). Briefly, according to the manufacturer's protocol, DNA samples from control and E2-stimulated HMA-1 and LAD2 cells were first incubated with methylation-dependent restriction enzymes. A subsequent qPCR on prefabricated 96-well plates and application of the appropriate software (Qiagen) enabled the quantification of individual CpG methylation levels. The qPCRs were done using the RotorGene SYBR Green PCR Kit (Qiagen) and the CFX96 Touch™ RealTime PCR Detection System (Biorad). Additionally, HMC-1 and LAD2 cells were analyzed using pyrosequencing for CpG-promoter methylation of *CXCL12*, *CXCR4* and *IL-13* genes using appropriate primer sets (Qiagen) (Table 1, Supplementary Figure 3).

### Detection of mast cell tryptase in PCa and BPH tissue samples

Tissue samples from prostate carcinoma (PCa, obtained by radical prostatectomy) and benign prostate hyperplasia (BPH, obtained by transurethral resection of prostate) were collected at the Clinic of Urology, Pediatric Urology and Andrology, JLU Giessen, Germany. Patients gave their written informed consent for participation in a research study. The study was approved by the ethical committee of the Medical Faculty, JLU Giessen (ethical vote, AZ.: 123/12). Prostate tissue samples were characterized at the Institute of Pathology, JLU Giessen. Immunohistochemistry was performed on paraffin-embedded tissue sections. Briefly, after deparaffinization with Xylol, the tissue slides were washed with ethanol and cooked in antigen retrieval buffer (10% citrate, pH = 6.0). After permeabilization, slides were incubated at 4°C overnight with monoclonal anti-α-Tryptase (1:1000, Santa Cruz, sc-59587) in 2% PBT (PBS containing 2% Triton X-100). Then, slides were washed thrice with 2% PBT and incubated for 1 hour with biotinylated polyclonal goat α-rabbit IgG (1:200, Dako Denmark A/S, E0432). Development was done with VECTASTAIN Elite ABC-Peroxidase (Vector Laboratories) using 3,3′-diaminobenzidine (DAB) enhanced liquid substrate systems (Sigma-Aldrich). Tryptase positive mast cells were counted in PCa (*n* = 4) and BPH samples (*n* = 4) in tissue areas of the same size and mean values were compared using *t*-test.

### Statistical analysis

Statistical analyses were done with SPSS 23.0 (IBM) and Prism 7.02 (Graphpad). Median values are given for age, basic semen parameters, IPSS, CPSI scores and biochemical parameters. Median values and ranges (minimum-maximum) for mRNA and promoter CpG-methylation levels are given in Boxplots and shown in figures. Non-parametric variables were compared using the Mann–Whitney *U*-test (2-sided). Only *p*-values ≤ 0.02 were considered as statistically significant.

## SUPPLEMENTARY MATERIALS FIGURES



## Abbreviations

CP/CPPS: chronic prostatitis chronic pelvic pain syndrome
ESR1: estrogen receptor alpha
ESR2: estrogen receptor beta
E_2_: estradiol
AR: androgen receptor
PCa: prostate carcinoma
BPH: benign prostate hyperplasia
CPSI: chronic prostatitis symptom index
IPSS: international prostate symptom score
