# Supplementary material for: Elevated seminal plasma estradiol and epigenetic inactivation of ESR1 and ESR2 is associated with CP/CPPS
Source: Oncotarget. 2018 Apr 13;9(28):19623–39. doi: 10.18632/oncotarget.24714 (PMC5929413; doi:10.18632/oncotarget.24714)
Supplement: Supplementary file 1 [file oncotarget-09-19623-s001.pdf]

Elevated seminal plasma estradiol and epigenetic inactivation of *ESR1* and *ESR2* is associated with CP/CPPS

SUPPLEMENTARY MATERIALS

		Raw Values												Signifacance Values					
		HV <40 years			CP/CPPS <40 years			HV 40+ years			CP/CPPS 40+ years			HV vs. CP/CPPS	HV vs. CP/CPPS	HV vs. CP/CPPS	HV vs. HV	CP/CPPS vs. CP/CPPS	HV vs. CP/CPPS
		n	Median	STD	n	Median	STD	n	Median	STD	n	Median	STD	<40 years	<40 years (age-matched)	40+ years	<40 years vs. 40+ years	<40 years vs. 40+ years	all probands
	Age (Years)	30	25,55	5,29	26	31,41	5,43	31	48,00	8,03	24	48,10	6,28	0,007	0,936	0,270	<0.001	<0.001	0,272
Routine Semen Analysis	Volume (mL)	30	3,00	1,63	26	2,40	1,62	19	3,00	1,29	24	1,75	1,41	0,027	0,178	0,001	0,797	0,321	<0.001
	pH	30	8,10	0,31	26	7,60	0,29	19	7,40	0,21	24	7,50	0,38	<0.001	<0.001	0,673	<0.001	0,264	0,002
	Spermatozoa/mL	30	62,40	65,60	26	31,70	37,12	19	62,40	44,53	24	27,30	80,87	0,015	0,013	0,074	0,829	0,869	0,002
	Spermatozoa/Total	30	175,75	170,84	26	67,30	162,47	19	165,00	192,64	24	45,73	154,31	<0.001	0,002	0,001	0,790	0,749	<0.001
	Motility Grade A (Progressive)	30	47,50	19,30	26	21,50	15,05	19	5,00	12,77	21	13,00	9,83	<0.001	0,011	0,503	<0.001	0,174	0,004
	Motility Grade B (Non-linear)	30	17,00	8,42	26	24,00	9,40	19	29,00	11,65	21	25,00	9,07	0,003	0,069	0,153	<0.001	0,889	0,129
	Motility Grade C (Non-Progressive)	30	7,50	5,65	26	8,00	4,33	19	11,00	6,17	21	11,00	9,39	0,433	0,872	0,872	0,091	0,197	0,412
	Motility Grade D (Immotile)	30	27,50	14,34	26	37,00	16,55	19	43,00	15,21	21	44,00	14,05	0,005	0,069	0,282	0,001	0,023	0,003
	Vitality (Eosin Test)	30	82,00	7,54	8	68,00	17,07	17	73,00	13,19	9	68,00	20,17	0,013	0,121	0,164	0,006	0,742	0,001
	Peroxidase+ Cells (Mio/mL)	29	0,00	0,21	25	0,10	0,23	19	0,10	0,16	24	0,20	0,26	0,217	0,320	0,133	0,337	0,213	0,045
	MAR Test IgG	2	7,00	9,90	20	1,00	8,80	9	0,00	2,96	16	0,00	5,21	0,779	0,837	0,760	0,582	0,863	0,814
	MAR Test IgA	2	0,00	0,00	20	1,50	6,63	9	0,00	4,69	16	1,50	8,94	0,312	0,327	0,718	0,436	0,888	0,449
	Morphology Normal (%)	30	14,00	10,16	25	5,00	10,54	19	5,00	4,66	18	6,00	5,41	<0.001	0,001	0,753	<0.001	0,980	0,001
	Morphology Head Defects (%)	30	69,50	12,27	25	83,00	13,77	19	82,00	9,70	18	79,50	11,89	<0.001	0,001	0,258	<0.001	0,475	0,023
	Morphology Mid-Piece Defects (%)	30	49,00	16,42	25	6,00	13,99	19	13,00	7,83	18	12,00	7,71	<0.001	<0.001	0,443	<0.001	0,720	<0.001
	Morphology Tail Defects (%)	30	16,50	13,63	25	45,00	21,68	19	48,00	15,54	18	40,50	18,27	<0.001	<0.001	0,845	<0.001	0,971	<0.001
	Spermatogenese Cells (Mio/mL)	28	0,15	2,87	22	0,70	1,11	16	1,55	1,95	16	0,89	5,17	0,265	0,732	0,491	0,001	0,095	0,61
	Fructose (pg/mL)	5	15,10	5,56	24	16,35	10,42	19	15,20	8,55	22	17,30	8,60	0,594	0,489	0,592	0,945	0,495	0,425
	alpha-Glucosidase (pg/mL)	4	15,50	17,08	24	13,40	10,00	19	19,80	10,00	21	15,40	10,01	0,776	0,557	0,320	0,725	0,531	0,136
	Elastase (pg/mL)	4	11,00	46,99	24	20,00	382,71	19	79,00	111,38	21	98,00	159,86	0,547	0,456	0,893	0,116	0,088	0,72
	Zinc (pg/mL)	4	4,00	2,10	24	4,30	2,99	19	6,10	3,02	18	4,55	3,84	0,776	1,000	0,499	0,505	0,939	0,475
	IL-8 (pg/mL)	29	1835,00	2477,03	25	1888,00	3631,81	19	3249,00	2275,83	21	6079,00	7299,31	0,869	1,000	0,196	0,005	0,005	0,325
Blood Values	Testosterone (pg/mL)	0	N/A	N/A	25	458,00	288,11	0	N/A	N/A	24	315,00	140,03	N/A	N/A	N/A	N/A	0,010	N/A
	Estradiol (pg/mL)	30	34,50	11,61	22	36,50	10,97	18	42,00	5,71	23	35,00	11,51	0,753	0,165	0,017	0,054	0,440	0,105
	CRP (pg/mL)	0	N/A	N/A	24	0,00	7,73	0	N/A	N/A	24	0,90	6,49	N/A	N/A	N/A	N/A	0,004	N/A
	PSA (pg/mL)	0	N/A	N/A	23	0,57	0,31	0	N/A	N/A	23	0,79	1,26	N/A	N/A	N/A	N/A	0,132	N/A
Uro-flow	Maximum Flowthrough (mL/sec)	0	N/A	N/A	24	18,60	7,67	0	N/A	N/A	20	14,75	7,61	N/A	N/A	N/A	N/A	0,258	N/A
	Total Volume (mL)	0	N/A	N/A	24	340,00	189,28	0	N/A	N/A	20	290,50	125,43	N/A	N/A	N/A	N/A	1,000	N/A
Questionnaires	CPSI Pain	25	0,00	2,00	23	12,00	4,37	0	N/A	N/A	24	13,50	2,93	<0.001	<0.001	N/A	N/A	0,104	<0.001
	CPSI Urinary	25	1,00	1,45	23	4,00	2,64	0	N/A	N/A	24	5,00	2,51	<0.001	<0.001	N/A	N/A	0,198	<0.001
	CPSI Quality of Life	25	0,00	1,48	23	8,00	2,95	0	N/A	N/A	24	10,00	1,79	<0.001	<0.001	N/A	N/A	0,113	<0.001
	CPSI Total	25	1,00	3,69	23	24,00	8,41	0	N/A	N/A	24	28,00	4,78	<0.001	<0.001	N/A	N/A	0,115	<0.001
	IPSS	19	2,00	2,24	23	11,00	6,34	0	N/A	N/A	24	15,00	6,09	<0.001	<0.001	N/A	N/A	0,016	<0.001
	HADS Anxiety	7	5,00	2,45	13	7,00	3,75	0	N/A	N/A	11	7,00	6,23	0,056	0,077	N/A	N/A	0,910	N/A
	HADS Depression	9	2,00	4,47	13	8,00	3,78	7	7,00	3,78	11	5,00	4,99	0,001	0,049	1,000	N/A	1,000	0,095
	HADS Total	7	7,00	3,44	13	16,00	7,09	0	N/A	N/A	11	12,00	10,89	0,003	0,028	N/A	N/A	0,733	N/A

Supplementary Figure 1: The summary of clinical data obtained from CP/CPPS patients and healthy donors. The raw data comprise semen parameters (available for all probands), biochemical parameters of ejaculates (available for all probands), biochemical parameters of blood (available for CP/CPPS patients only), uroflow parameters (available for CP/CPPS patients only), data collected in the frame of CP/CPPS relevant questionnaires (available for all CP/CPPS patients and for young healthy donors). Statistical comparison for each of the available parameters was done with regard to the age of the probands in order to distinguish between age-effects and disease (CP/CPPS)-effects (highly significant differences, $p \leq 0.01$, are shown in dark red, and not significant differences are shown in steps from light red to green). Abbreviations: HV: healthy volunteers; CP/CPPS: chronic prostatitis chronic pelvic pain syndrome; n: number of analyzed probands; STD: standard deviation; N/A: not applicable; IL-8: interleukin 8; CRP: C-reactive protein; PSA: prostate specific antigen; CPSI: chronic prostatitis symptom index; IPSS: international prostate symptom score; HADS: hospital anxiety and depression score.

	Gene Symbol	Full Gene Name	Fold Regulation (HMC-1)	Fold Regulation (LAD2)
Interleukin Family Genes	IL1B	interleukin 1 beta	-2.8	1.3
	IL6	interleukin 6	-2.2	1.7
	IL7	interleukin 7	-1.1	-2.8
	IL8	interleukin 8	-2.4	30.6
	IL9	interleukin 9	-2.1	-1.1
	IL12B	interleukin 12B	-2.2	2.3
	IL16	interleukin 16	1.3	-6.1
	IL17F	interleukin 17F	-2.9	3.7
	IL21	interleukin 21	-1.4	4.9
	IL22	interleukin 22	-1.8	5.4
	IL27	interleukin 27	-2.1	2.3
C-Chemokine Family Genes	CCL5	C-C motif chemokine ligand 5	-2.9	1.5
	CCL7	C-C motif chemokine ligand 7	-1.7	3.5
	CCL8	C-C motif chemokine ligand 8	-1.0	2.3
	CCL13	C-C motif chemokine ligand 13	-1.9	2.1
	CCL18	C-C motif chemokine ligand 18	-7.3	1.5
	CCL20	C-C motif chemokine ligand 20	-2.1	1.2
	CX3CL1	C-X3-C motif chemokine ligand 1	-1.1	2.8
	CXCL2	C-X-C motif chemokine ligand 2	-9.5	6.5
	CXCL11	C-X-C motif chemokine ligand 11	-2.1	-1.1
	CXCL16	C-X-C motif chemokine ligand 16	-2.5	1.1
	XCL1	X-C motif chemokine ligand 1	-9.4	-1.1
Other Genes	BMP2	bone morphogenetic protein 2	-3.1	1.0
	BMP7	bone morphogenetic protein 7	-6.6	-2.4
	CSF3	colony stimulating factor 3	-3.6	4.3
	IFNA2	interferon alpha 2	3.7	2.3
	IFNG	interferon gamma	3.8	4.4
	MSTN	myostatin	-1.3	4.9
	SPP1	secreted phosphoprotein 1	-2.3	-1.1
	TNFRSF11B	TNF receptor superfamily member 11b	-2.6	-1.1
	TNFSF11	tumor necrosis factor superfamily member 11	-2.5	-1.8
	VEGFA	vascular endothelial growth factor A	-2.0	1.3

Supplementary Figure 2: Summary of transcriptional analyses of inflammatory factors in mast cell lines before and after estradiol treatment. Human mast cell lines HMC-1 and LAD2 were treated with 100 nM estradiol (E_2) for 24 hours. RNA samples from control (untreated) and E_2-treated cells were reverse transcribed and analyzed using RT2 Profiler PCR array "Human cytokines and chemokines" (Qiagen, covers 84 inflammatory genes). Expression data of detectable genes are presented as a fold regulation in comparison to controls. Upregulation and downregulation ("-") values more than 2-fold are highlighted in red and blue, respectively. HMC-1 cells, which possess through CpG-promoter methylation inactivated estrogen receptors, *ESR1* and *ESR2*, responded to E_2-excess with more than 2-fold downregulation of 21 out of 32 detectable inflammatory factors (7 genes exhibited unchanged transcription levels and 2 genes were upregulated). In contrast, LAD2 cells possessing unmethylated and active *ESR1* and *ESR2*, responded to E_2 with upregulation of 15 out of 32 detectable inflammatory factors (14 genes exhibited unchanged transcription levels and 3 genes were downregulated). Abbreviations: ILs: interleukins; CCLs: C-C motif chemokines; CXCLs: C-X-C motif chemokines; BMPs: bone morphogenetic proteins; CSF3: colony stimulating factor 3; IFNs: interferons; MSTN: myostatin; SPP1: secreted phosphoprotein 1; TNFRSF11B: tumor necrosis factor receptor superfamily member 11B; TNFSF11: tumor necrosis factor superfamily member 11; VEGFA: vascular endothelial growth factor alpha.

		LAD2 [%]		HMC-1 [%]		Methylation Change [%]	
Method	Gene	Control	100nM E2	Control	100nM E2	LAD2	HMC-1
Methyl II Array	BCL10	0	0	3	1	0	-2
	BCL3	0	0	0	61	0	61
	ELANE	100	100	99	99	0	0
	FOXP3	99	99	99	98	0	-1
	GATA3	51	1	0	0	-50	0
	HMOX1	1	1	0	0	0	0
	IGF2BP2	0	0	93	85	0	-8
	IL12A	1	1	0	1	0	1
	INHA	66	28	72	70	-37	-2
	INHBA	100	100	100	100	0	0
	IRF1	0	0	0	0	0	0
	LTB	86	85	N/A	N/A	-2	N/A
	MALT1	N/A	0	1	N/A	N/A	N/A
	MAP3K7	0	0	1	6	0	5
	MYD88	0	0	0	0	0	0
	NOD1	0	0	N/A	0	0	N/A
	SMAD3	100	100	100	99	0	-1
	SOD1	0	0	1	0	0	-1
	STAT5A	82	81	2	2	-1	0
	TLR2	0	0	0	56	0	56
	TRAF2	0	0	0	0	0	0
	TRAF6	0	0	0	0	0	0
Pyrosequencing	CXCL12	74	74	75	77	0	2
	ER1	2	2	68	67	0	-1
	ER2	3	2	94	93	-1	-2
	AR(01)	2	2	44	43	0	0
	AR(04)	4	3	42	41	-1	0
	IL-13	39	38	20	17	-1	-4
	CXCR4(01)	1	1	1	1	0	0
	CXCR4(05)	2	2	2	1	0	-1

Supplementary Figure 3: Summary of CpG-promoter methylation analyses of inflammatory factors in mast cell lines before and after estradiol treatment. DNA from untreated (control) and estradiol treated (100 nM, 24 hours) mast cell lines HMC-1 and LAD2 was bisulfite-converted and analyzed using EpiTect Methyl II Signature PCR array "Human cytokine production" (Qiagen, covers 22 inflammatory genes) and by pyrosequencing (6 genes) with ready-to-use primer sets (Qiagen) for indicated genes (note: *AR* and *CXCR4* were analyzed in two different promoter regions). High levels of promoter methylation were found in control LAD2 cells in 10 genes and in control HMC-1 cells in 11 (indicated in green). For most inflammatory genes, E_2 treatment did not have an effect on DNA methylation. However, four out of 28 analyzed genes showed significant changes (50% and 37% decrease of methylation in LAD2 cells in *GATA3* and *INHA*, respectively; 61% and 56% increase of methylation in HMC cells in *BCL3* and *TLR2*, respectively). Abbreviations (gene with noticeable methylation and methylation changes are listed): BCL3: B-cell CLL/Lymphoma 3; ELANE: elastase, neutrophil expressed; FOXP3: forkhead box P3; GATA3: GATA binding protein 3; IGF2BP2: Insulin-like growth factor 2 mRNA-binding protein 2; INHA: inhibin A; INHBA: inhibin beta A subunit; LTB: lymphotoxin beta; SMAD3: SMAD family member 3; STAT5A: Signal transducer and activator of transcription 5A; TLR2: toll-like receptor 2; CXCL12: C-X-C motif chemokine 12; ER1: estrogen receptor alpha; ER2: estrogen receptor beta; AR: androgen receptor; IL13: interleukin 13; N/A: not applicable as did not pass the quality control.
